# The Development of a Sorghum Bran-Based Biorefining Process to Convert Sorghum Bran into Value Added Products

**DOI:** 10.3390/foods8080279

**Published:** 2019-07-24

**Authors:** Oyenike Makanjuola, Darren Greetham, Xiaoyan Zou, Chenyu Du

**Affiliations:** 1 School of Applied Sciences, University of Huddersfield, Queensgate, Huddersfield HD1 3DH, UK; 2 Key Laboratory of Functional Inorganic Material Chemistry, Heilongjiang University, Harbin 150080, China

**Keywords:** glucoamylase, sorghum milling waste, submerged fungal fermentation, *Aspergillus awamori*, hydrolysis, waste valorization

## Abstract

Sorghum bran, a starch rich food processing waste, was investigated for the production of glucoamylase in submerged fungal fermentation using *Aspergillus awamori*. The fermentation parameters, such as cultivation time, substrate concentration, pH, temperature, nitrogen source, mineral source and the medium loading ratio were investigated. The glucoamylase activity was improved from 1.90 U/mL in an initial test, to 19.3 U/mL at 10% (w/v) substrate concentration, pH 6.0, medium loading ratio of 200 mL in 500 mL shaking flask, with the addition of 2.5 g/L yeast extract and essential minerals. Fermentation using 2 L bioreactors under the optimum conditions resulted in a glucoamylase activity of 23.5 U/mL at 72 h, while further increase in sorghum bran concentration to 12.5% (w/v) gave an improved gluco-amylase activity of 37.6 U/mL at 115 h. The crude glucoamylase solution was used for the enzymatic hydrolysis of the sorghum bran. A sorghum bran hydrolysis carried out at 200 rpm, 55 °C for 48 h at a substrate loading ratio of 80 g/L resulted in 11.7 g/L glucose, similar to the results obtained using commercial glucoamylase. Large-scale sorghum bran hydrolysis in 2 L bioreactors using crude glucoamylase solution resulted in a glucose concentration of 38.7 g/L from 200 g/L sorghum bran, corresponding to 94.1% of the theoretical hydrolysis yield.

## 1. Introduction

The increasing concerns about global energy shortages and environmental pollution have encouraged research on the development of biorefining strategies for the conversion of renewable raw materials into value added products. Various crops, such as wheat, corn and rapeseed, that are historically only used for food, however, have now been targeted as starting materials for the production of biofuels, biochemical and biopolymers. Sorghum (*Sorghum bicolor*) is a cereal plant of the grass family *Gramineae* which originates from Africa [1]. It is the 5th most important crop cereal in the world in terms of its acreage and production [2]. Sorghum is particular important to African countries, as it grows well in hot and arid regions [3]. Traditionally, sorghum grains are cleaned, conditioned, tempered and debranned in grain hulliers to remove the outermost fibrous layer. It is then milled and sieved (dry milling process) to obtain a flour fraction using a Buhler mill [4].

**Figure 1:**
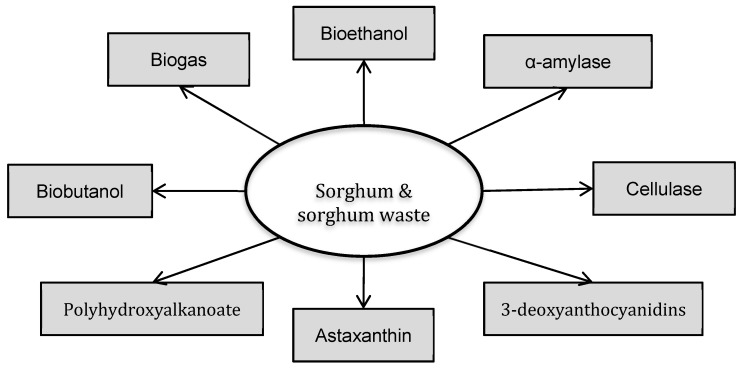
The schematic diagram showing the potential food ingredient and non-food products that can be derived from sorghum and sorghum waste.

Besides direct human food application, sorghum crops have been used in many fields, such as animal feed, and the production of biofuels, enzymes and bioactive compounds. Figure 1 shows a schematic diagram of sorghum-based biorefinery approaches for the production of various non-food products. Biofuel is one of the fields in which sorghum has received significant attention. Li et al. reported a demonstration study of converting sweet sorghum stems into bioethanol [5]. Sixteen tons of sweet sorghum stems were used and 1 ton of ethanol (99.5% v/v) was obtained. The cost of sorghum-derived fuel bioethanol was estimated to be $0.49 per litre [5]. It was estimated that the sweet sorghum production in China using only marginal land could reach 13.57 million tons [6]. Approximately 0.85 million tons of bioethanol could be produced [6]. Ahmed El-Iman et al. estimated the bioethanol production potential in Nigeria using sorghum bran [7], reaching the conclusion that 497 million US gallons of bioethanol could be produced, which equivalent to 17% of Nigeria’s transportation fuel use. Beside bioethanol, sorghum has also been investigated for biobutanol [8], biogas [9] and biohydrogen [10] production. As for high value markets, sorghum has been reported as the basis for the synthesis of astaxanthin [11], 3-deoxyanthocyanidins and various other bioactive compounds [12]. Lolasi et al. 2018 cultivated a halotolerant bacterium *Nesterenkonia sp* on sorghum bagasse hydrolysate and obtained an α-amylase solution of 97 U/mL [13], demonstrating the feasibility of using sorghum waste streams to produce enzymes. These studies have suggested that sorghum and sorghum processing waste have a huge potential for the production of value added products.

Glucoamylase is an important enzyme for starch hydrolysis due to its catalytic effect of releasing glucose from the non-reducing ends of starch [14]. It is widely used in the food, feed and pharmaceutical industries, mainly for the production of glucose syrup, high fructose corn syrup, and alcohol. Traditionally, filamentous fungi were used for producing glucoamylase, with *Aspergillus niger, Aspergillus awamori* and *Rhizopus oryzae* being the major strains used for commercial gluco-amylase production [15]. 

To reduce the production costs, various agriculture residues have been explored for gluco-amylase production, including wheat bran, green gram bran, black gram bran, corn flour, barley flour, maize bran, rice bran, rice flakes and food waste. Table 1 lists some recent studies on gluco-amylase production together with the fermentation conditions used in these reports. 

Media composition and growth conditions were reported to influence glucoamylase production significantly. At low concentrations, glucose has been reported to be an inhibitor for the production of glucoamylase, while some nitrogen sources such as yeast extract, ammonium sulphate, ammonium nitrate, urea, meat extract and peptone have been used to promote glucoamylase production [28,29]. Optimization of fermentation either using one factor at a time design [20,22,23] or using response surface method [24] is still a main approach to improve glucoamylase production, along with gluco-amylase producing strain selection and genetic modification [20,24]. It has been shown that a 24% increase in glucoamylase activity was achieved through optimization of SSF media and parameters by *A. oryzae* using agro residues as substrate [30]. 

Although sorghum bran is one of the major agriculture waste streams, and contains high residual starch (up to 53% (w/w) [7]), the utilization of sorghum bran has not been fully investigated. In this study, a sorghum bran-based biorefining concept has been developed for glucoamylase production and using the resulting glucoamylase to hydrolyze sorghum bran to produce a sugar-rich generic fermentation medium. Glucoamylase synthesis using *Aspergillus awamori* via Submerged Fermentation (SmF) was carried out. Fermentation conditions, such as substrate concentration, pH and temperature were investigated using one factor at a time design. Bench top fermentation for relatively large-scale enzyme production was carried out to produce sufficient amount of crude enzyme, which was subsequently used for the hydrolysis of sorghum bran and compared with commercial enzymes. Finally, the economic benefit of utilizing sorghum bran for the production of glucoamylase was discussed briefly. 

## 2. Materials and Methods

### 2.1. Sorghum Bran

The sorghum (*Sorghum bicolor*) used in this study is a variety of red sorghum, which was purchased from a local market in Ikorodu, Lagos State, Nigeria in Spring 2017. The sorghum was subjected to three different milling processes using smart peanut butter maker (wet milling), blender (wet milling) and knife mill (dry milling). For wet milling, sorghum was steeped in tap water (2:5 w/v) for 3 days at room temperature and was wet milled using either a smart peanut butter maker (Smart^@^, Nostalgia, Amazon, UK) or a food-processing blender (Cookworks, Argos, UK ). The milled biomass was sieved with muslin cloth to remove the starch component from the slurry. The remaining component sorghum bran (consisting of the outer layers of the cereal grain and residual starch) was dried in an oven at 60 °C for 3 days. For milling using a knife mill, air dried sorghum grain was milled by a lab knife mill to pass through a 2-mm sieve screen. The resulting product was then subsequently sieved using a 1-mm sieve. The particles above 1-mm were used as sorghum bran. The total starch content of the sorghum bran sample was determined using an enzymatic starch analysis kit (Megazyme^®^, Bray, Ireland). 

### 2.2. Microorganisms 

The strain used for glucoamylase production was *Aspergillus awamori* 2B.361 U2/1 (*A. awamori*), which was kindly provided by Prof. Colin Webb, the University of Manchester (UK). Procedures for storing, and cultivating *A. awamori* were described by Koutinas et al. [31]. 

### 2.3. Sorghum Bran Submerged Fermentation 

Submerged fermentation was carried out both in shake flask for preliminary assessment and in 2-L fermenters (Electrolab FerMac 360, UK) for large-scale enzyme production. The sorghum bran concentration used in the initial fermentation was 4% (w/v). 250 mL shaking flasks were used in all experiments except the study on the impact of medium loading ratio. The working volume was 100 mL unless specified. Several drops of silicon antifoam (0.002% v/v) were added to the complex medium in order to prevent foaming. Unless specified, no other nutrient or chemical were added into the fermentation media. The media were sterilised at 121 °C for 15 min and allowed to cool down before *A. awamori* was added at an inoculation ratio of 1 × 10^7^ spores/g dry weight sorghum bran. The mixture was fermented in a shaking incubator (Incu-Shake FL24-1R, SciQuip, UK) at 28 °C and 200 rpm. Glucoamylase production was investigated using different conditions, including initial pH (3.0–8.0), temperature (26 °C, 28 °C and 30 °C), substrate concentration (2–10% w/v), medium loading ratio (50–250 mL in 500 mL bottle), yeast extract (0–10 g/L, Sigma-Aldrich, UK), and with and without the addition of minerals. Five mineral solutions were explored in this study, as shown in Table 2. All SmF were carried out in triplicates. 

For large-scale glucoamylase production, strain *A. awamori* was cultured in 250 mL shaking flasks containing 50 mL inoculation medium. The inoculation medium contained 40 g/L of glucose and 10 g/L of yeast extract. The seed fermentation was carried out at 28 °C, 200 rpm for 3 days in the shaking incubator (SciQuip Incu-Shake FL24-1R). The fermentation medium contained 10% (w/v) sorghum bran, 2.5 g/L yeast extract and was prepared with mineral solution C. 1 mL of sterilized silicon antifoam (0.02%, v/v) was added to the fermentation medium before inoculation. The fermentation was carried out at 28 °C, 500 rpm and an aeration rate at 1.0 L/min. The pH was controlled to 6.0 by adding 2 M NaOH solution or 2 M HCl solution. 10 mL of sample were taken at different time intervals for glucoamylase analysis.

### 2.4. Glucoamylase Enzyme

Glucoamylase activity was measured using the method described by Bernfeld [35]. Two mL of fermentation sample was spun in a centrifuge (Eppendorf, UK) at 5000 rpm for 5 min to remove cell. The liquid suspension was then used as crude enzyme solution. A reaction mixture containing 0.9 mL of 0.05 mM citrate buffer (pH 5.0), 1.0 mL autoclaved starch solution (1%, w/v) and 0.1 mL of crude enzyme solution was incubated at 50 °C for 20 min. Then 3 mL of 3,5-dinitrosalicyclic acid (DNSA) reagent [36] was added to the incubated mixture. The reaction mixture was heated in a vigorously boiling water bath for 5 min and was allowed to cool. Absorbance was measured using a spectrophotometer at 540 nm. Glucoamylase activity unit (U) was express as the amount of enzyme releasing one µmole of glucose equivalent per minute under the assay condition and enzyme activity was express in terms of units per mL (U/mL).

### 2.5. Enzymatic Hydrolysis of Sorghum Bran

The enzymatic hydrolysis of sorghum bran was initiated by gelatinizing a mixture of 4 g sorghum bran in 50 mL of deionised water in a boiling water bath at 100 °C for 20 min in a 250 mL conical flask. The agitation was carried out using a glass rod to mix for 30 s every 5 min. After gelatinisation, the substrate was cooled to 55 °C. Then, either crude gluco-amylase enzyme solution or commercial enzymes (gluco-amylase and α-amylase from Megazyme^@^) were added at an enzyme loading ratio of 50 U/g. The hydrolysis was carried out in a shaking incubator (SciQuip Incu-Shake MIDI), 200 rpm at 55 °C for 48 h. The sorghum bran hydrolysis yield was calculated using the following equation:

(1) $$Hydrolysis\;yield=\frac{Weight\;of\;glucose\;}{Weight\;of\;bran\times starch\;content\times 1.11}$$

### 2.6. Sugar Analysis

The amounts of sugars were quantified by HPAEC-PAD. The sample was filtered through a 0.2 µm syringe filter and was then transferred into a 1.5 mL agilent auto sampler vial. The monosaccharides were analysed using a Dionex ICS-3000 Reagent-Free^TM^ Ion Chromatography on a with Dionex ICS-3000 system, electrochemical detection using ED 1 and computer controller. A CarboPacTM PA 20 column (3 × 150 mm/; Dionex, Sunnyvale, CA, USA) was used and the mobile phase was 10 mM NaOH with a flow rate of 0.3 mL/min. The injection volume was 25 μL and the column temperature was 30 °C. 

### 2.7. Statistical Analysis 

Microsoft Excel (2013) was used to calculate the results obtained from all the experiment such as the standard deviation. 

## 3. Results and Discussion

### 3.1. Starch Content in the Sorghum Brans 

Three milling processes using a peanut butter maker (wet milling), blender (wet milling) and knife mill were examined for separating starch from sorghum bran. The starch contents of the resulting sorghum bran were analyzed as shown in Table 3. 

This revealed that extremely high starch (81.93%, w/w) was retained in the bran obtained after knife mill (dry milling), indicating that dry milling of sorghum grain in the lab was not suitable for separating the bran from the kernel. By contrast, wet milling using a peanut butter maker and the blender mill successfully isolated bran from sorghum kernel, resulting in sorghum brans containing only 16.4% and 13.0% starch, respectively. In comparison with other reports of starch composition in sorghum bran (Table 3), the starch concentrations obtained in this study were much lower, suggesting wet milling using a peanut butter maker or a blender was a suitable technology for sorghum starch recovery.

### 3.2. Glucoamylase Production Using Sorghum Bran Via SmF

**Figure 2:**
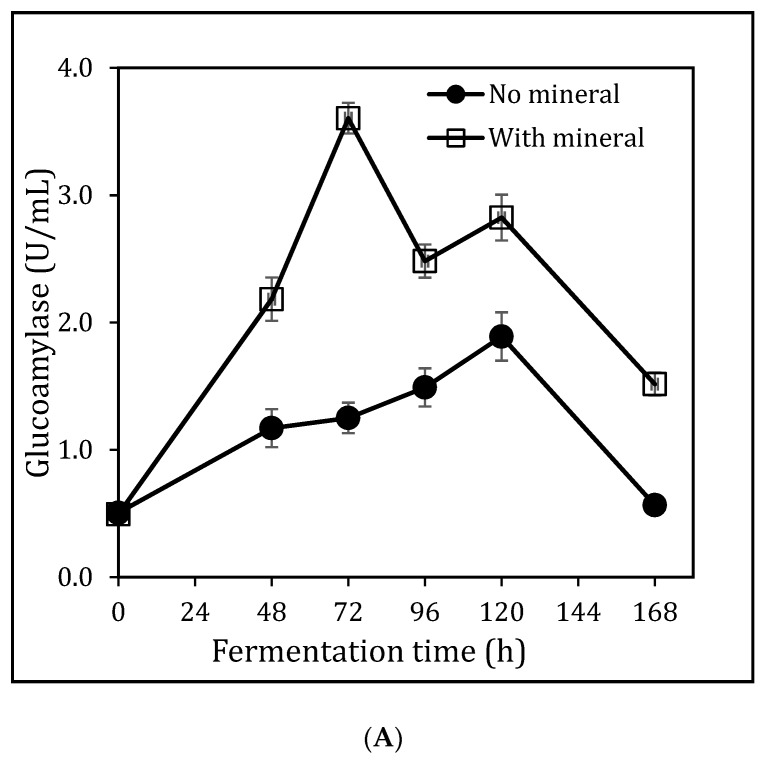

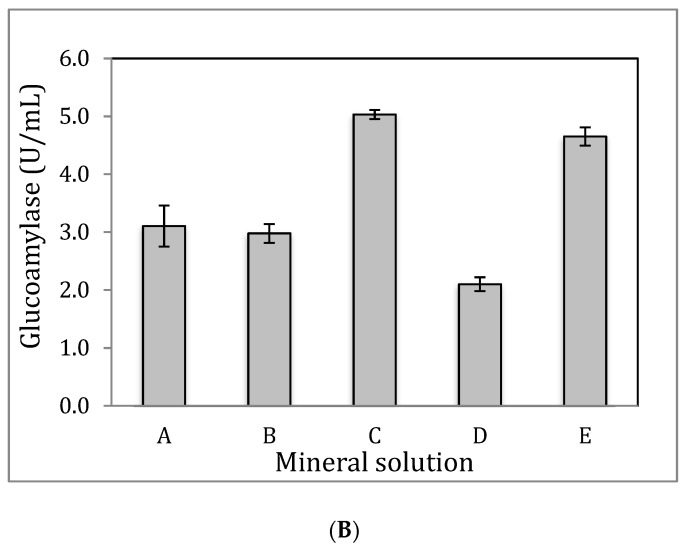
The effect of mineral solution on glucoamylase production in SmF. (**A**): the comparison of gluco-amylase production profile in SmF with and without mineral solution E. (**B**): Gluco-amylase production using mineral solutions A–E, fermentation time 72 h, temperature 28 °C, 4% (w/v) sorghum bran, no extra nutrients, no pH adjustment.

The wet milled sorghum bran derived using the peanut butter maker was used for the production of glucoamylase in a submerged fermentation (SmF). The initial fermentation was carried out using 4% (w/v) sorghum bran and no addition of nutrients. As shown in Figure 2A, an increase in glucoamylase activity was detected until 120 h, then the enzyme activities decreased sharply. The peak glucoamylase activity was 1.90 U/mL. 

To improve glucoamylase production, the sorghum bran was augmented with a mineral solution, containing K_2_HPO_4_ 2.5 g/L, NH_4_NO_3_ 1.5 g/L, KH_2_PO_4_ 1.5 g/L, MgSO_4_ 0.12 g/L and NaCl 0.25 g/L (Mineral solution E in Table 2), respectively. Adding mineral to the sorghum bran-containing fermentation medium increased the glucoamylase activity from 1.90 U/mL to 3.60 U/mL and reduced the time requirement for the peak enzymatic activity from 120 h to 72 h (Figure 2A). Further to this result, the supplement of four more mineral solutions were explored (Table 2), and the results are shown in Figure 2B. The mineral solution C led to the highest glucoamylase activity of 5.03 U/mL, which was then used in the following fermentation experiments. Comparing mineral solution C and mineral solution D, the removal of CaCl_2_ led to a significant decrease of glucoamylase activity, indicating that the calcium may play a key role in glucoamylase production. However, further experiment is required to confirm this hypothesis. 

### 3.3. Impact of Substrate Concentzration and pH on Glucoamylase Production

**Figure 3:**
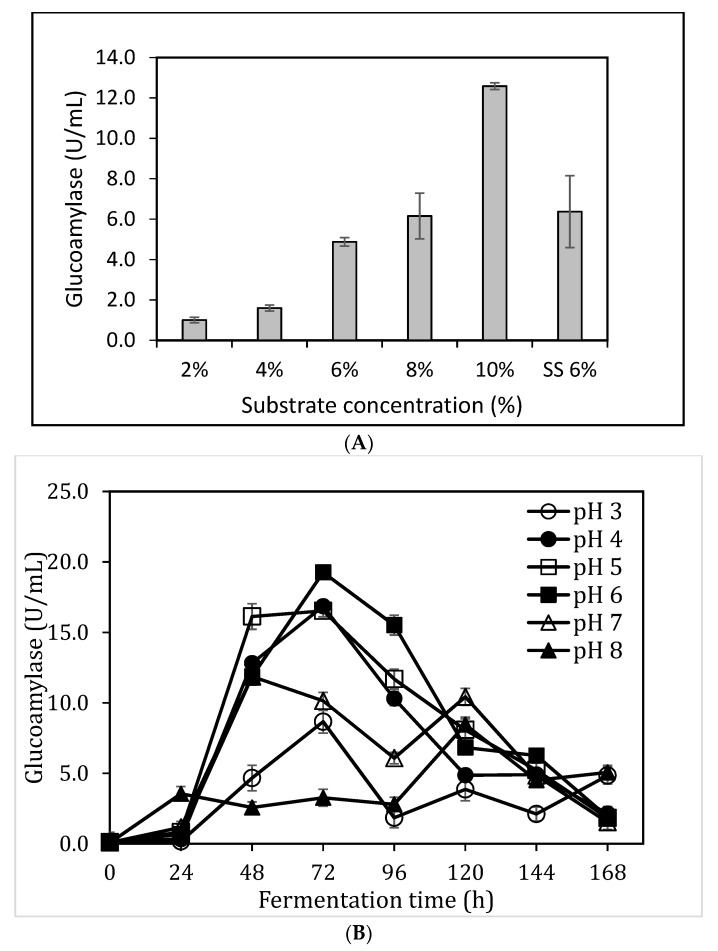
The effect of substrate concentration (**A**) and pH (**B**) on glucoamylase production. For the fermentation in (**3A**): fermentation time 72 h, temperature 28 °C, with mineral solution C, no extra nutrients, no pH adjustment. For the fermentation in 3B: temperature 28 °C, 10% (w/v) sorghum bran, with mineral solution C, no extra nutrients, no pH adjustment.

The substrate concentration determines the availability of carbon source in the fermentation system. The low glucoamylase activities observed in the above experiment could be due to an insufficient supply of carbon sources. The effect of sorghum bran concentration on glucoamylase activities in SmF was explored (2%, 4%, 6%, 8%, and 10%, w/v). The utilisation of 6% starch-rich sorghum milling product (SS) from peanut maker milling process was also included as a comparison. The starch-rich sorghum milling product contained 49.4% (w/w) starch, corresponding to ~18% sorghum bran concentration based on the total starch content. As shown in Figure 3A, glucoamylase activity increased as the substrate concentration increased. The highest glucoamylase activity of 12.6 ± 0.2 U/mL was obtained when 10% sorghum bran was used. The starch-rich sorghum milling product at 6% resulted in a similar glucoamylase activity (6.4 ± 1.8 U/mL) as that observed with 8% sorghum bran (6.2 ± 1.1 U/mL). These results indicated that sorghum bran contained some nutrients other than starch to facilitate glucoamylase synthesis. Further increasing substrate concentration was not carried out mainly due to the difficulty of mixing problem at high sorghum bran concentration in shake flasks. 

The effect of initial pH on glucoamylase production was investigated in SmF as shown in Figure 3B. A gradual increase in enzyme production was observed at pH 3.0, 4.0, 5.0 and 6.0, peak enzyme production obtained was 8.7 ± 0.8 U/mL, 16.9 ± 0.3 U/mL, 16.5 ± 0.4 U/mL, and 19.3 ± 0.5 U/mL, respectively. At pH 7, the peak enzyme activity was obtained at 48 h (11.9 ± 0.4 U/mL) before a declining trend was observed. There was a slow increase in glucoamylase activity at pH 8.0 for 96 h before a peak enzyme activity was observed at 120 h (8.5 ± 0.5 U/mL). 

The optimum pH for glucoamylase enzyme production was determined to be pH 6.0 after 72 h of the fermentation. These results agreed with previous results that 3 days of cultivation led to a better glucoamylase accumulation. Although a higher amylase synthesis was reported at pH 8.0 by *Bacillus sp* under SSF [38], the majority of research has reported that the best pH for amylase production was approximately pH 6.0 [39,40]. 

### 3.4. Impact of Medium Loading Ratio on Glucoamylase Production

The fermentation medium loading ratio affected the dissolved oxygen level and the mixing pattern in the shake flask. The dissolved oxygen level had an important impact on the physiology and metabolism of the microorganism. At a low level of oxygen supply, the production of essential enzymes was inhibited, while at a high aeration rate could have detrimental effects on the growth of microorganism and subsequent enzyme production. Mechanical mixing in a bioreactor determined the heat and mass transfer rates, therefore, impacting cell growth and enzyme synthesis. The impact of medium loading ratio on glucoamylase production was determined in SmF using 500 mL shake flasks at 200 rpm, 28 °C. 

**Figure 4:**
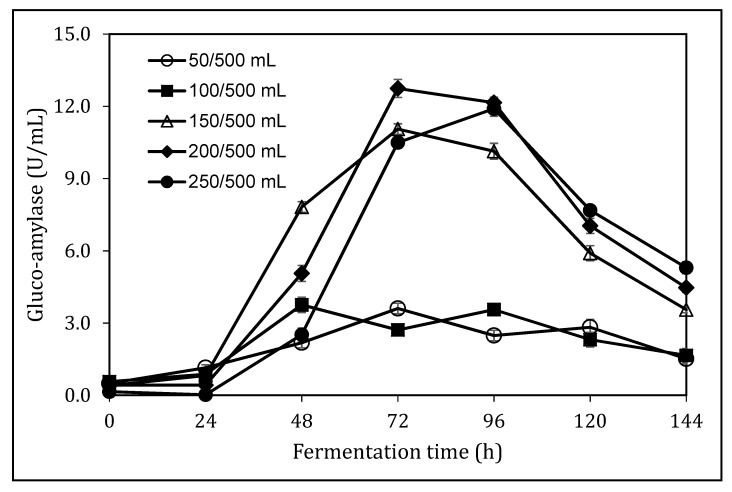
The effect of medium loading ratio on glucoamylase production. Fermentation was carried out at 28 °C, 10% (w/v) sorghum bran, with mineral solution C, at pH 6.0.

As shown in Figure 4, there was no significant increase in glucoamylase production when the aeration ratios of 50/500 mL or 100/500 mL were used. There was an increase in glucoamylase activity and a peak increase was obtained after 72 h of fermentation when 150/500 mL and 200/500 mL (11.1 ± 0.2 U/mL and 12.7 ± 0.3 U/mL) was used, respectively, while a peak increase in glucoamylase was obtained at 96 h when medium loading ratio of 250/500 mL (11.9 ± 0.3 U/mL) was used. It was expected that a low medium loading ratio would encourage oxygen transfer and thus benefit gluco-amylase production. However, the results in Figure 4 clear indicated a higher loading ratio at 200/500 mL was a preferred condition. This could attribute to the high viscosity in sorghum bran derived medium, which created significant mixing difficulty when the actual reaction volume was low. The insufficient mixing subsequently affected cell growth and glucoamylase production. 

### 3.5. Impact of Yeast Extraction and Temperature on Glucoamylase Production

**Figure 5:**
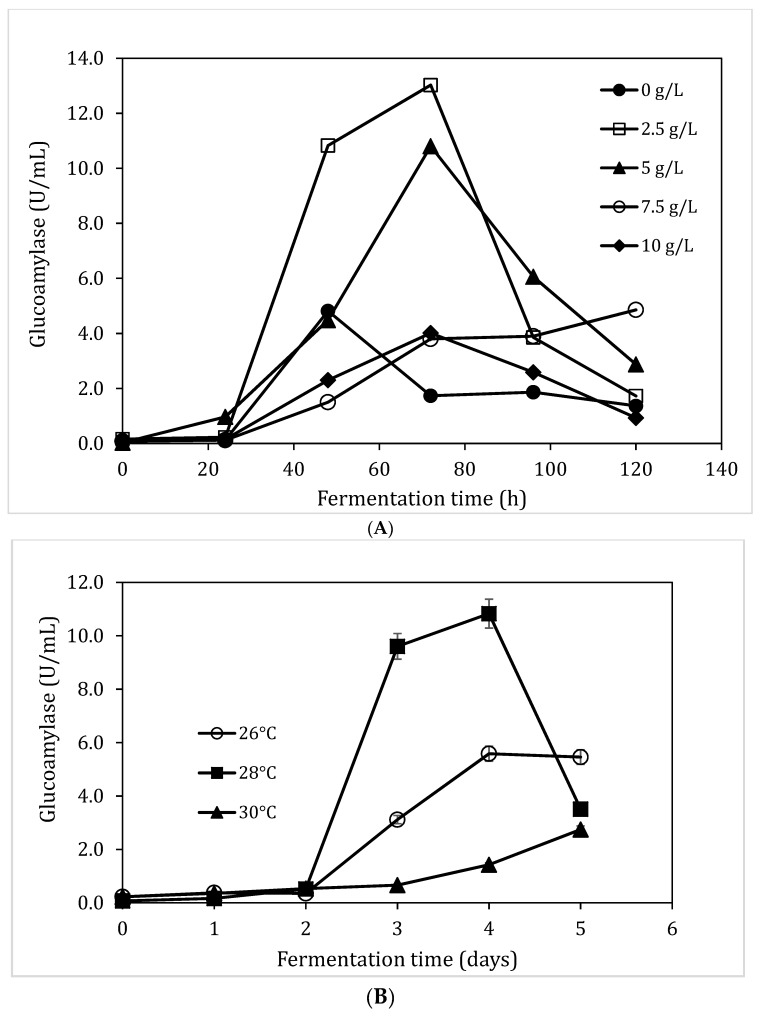
The effect of yeast extract (**A**) and temperature (**B**) on glucoamylase production in SmF. Fermentation was using 10% (w/v) sorghum bran, with mineral solution C at pH 6.0.

Yeast extract is used as a nitrogen and nutrient source in many bacterial culture media. Yeast extract contains an abundance of vitamins, minerals and amino acids, which are necessary for cell growth and enzyme synthesis. The addition of yeast extract was carried out with the aim to further increase glucoamylase enzyme production (Figure 5A). The optimum glucoamylase activity (13.0 ± 0.3 U/mL) was obtained after 3 days of fermentation with 2.5 g/L yeast extract addition. 

Temperature has an important effect on enzyme production, as a reaction rate generally increases with temperature to a maximal level before a decline occurs with further increase in temperature due to the enzymes’ susceptibility to denaturation. The SmF was explored using temperatures ranging from 26 °C to 30 °C. As shown in Figure 5B, fermentation at 28 °C had the most significant glucoamylase activity after 96 h of fermentation (10.8 ± 0.5 U/mL). At 30 °C, the gluco-amylase activity showed a slow gradual increase up to 120 h, while at 26 °C the glucoamylase activity had a similar pattern as observed at 28 °C, but with less glucoamylase accumulation. This results obtained in this study agreed with Khan and Yadav [39], which reported an optimum α-amylase production at 28 °C for *A. niger.* Maximum amylase production by *A. niger* and *R. stolonifera* was achieved at 30 °C [41,42].

### 3.6. Glucoamylase Production in Bench Top Fermenters

**Figure 6:**
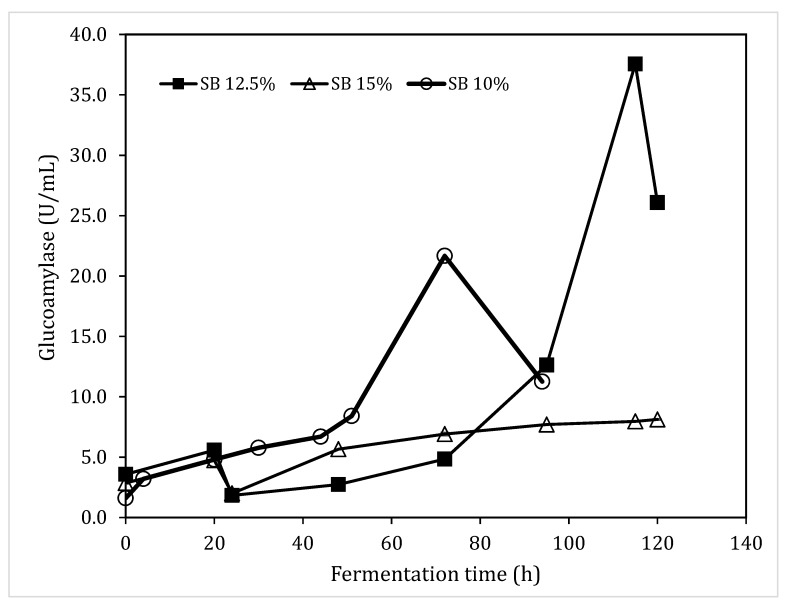
Typical glucoamylase accumulation profiles in 2-L fermenters using 10%, 12.5% and 15% (w/v) sorghum bran. Fermentation was carried out at 28 °C, 500 rpm, air aeration rate at 1.0 L/min, with mineral solution C at pH 6.0.

The above results indicated that the highest glucoamylase activity was achieved using a 10% (w/v) sorghum bran loading ratio, with addition of mineral solution C, 2.5 g/L yeast extract, at pH 6.0, 28 °C, and a liquid loading ratio of 200 mL in 500 mL shaking flasks. The fermentation conditions were used for the SmF of sorghum bran in 2-L fermenters. The scale up was repeated in four batches, and glucoamylase activities were in the range of 20.7 U/mL to 23.5 U/mL. A typical glucoamylase production profile was shown in Figure 6. Since bench top fermenters provide sufficient mixing at high substrate loading ratios, fermentations with high sorghum bran loading ratios of 12.5% and 15% (w/v) were also investigated. Typical glucoamylase production profiles are shown in Figure 6. When the substrate concentration was increased to 12.5%, glucoamylase production was enhanced to 37.6 U/mL (corresponding to 250 U/g dry weight sorghum bran), but the fermentation time was extended to 115 h. A sharp decline in enzyme activity at 120 h was observed due to foaming in the fermenter as a result of fungal autolysis. High stirring speed at 500 rpm was used due to high viscosity of the fermentation medium at high sorghum bran loading ratio. Vigorous agitation increased oxygen transfer and nutrient transfer, but resulted in mechanical stress, excessive foaming, disruption and physiological disturbance of cells. Further increasing substrate concentration to 15% did not lead to an improved glucoamylase production, mainly due to the insufficient mass transfer in the fermenter. 

The 2-L fermentation results confirmed that the optimum fermentation condition conditions obtained in shake flask experiments were valid. In comparison with the glucoamylase activities reported in the literature (Table 1), the glucoamylase activity of 250 U/g was close to that obtained using rice bran (264.5 U/g) [20], and was among the high glucoamylase activities reported. The results indicated that sorghum bran was a suitable substrate for the production of glucoamylase. Further more, the high enzyme concentration in the crude enzyme solution facilitates the following enzymatic hydrolysis, as it allows a higher concentration of substrate to used in the hydrolysis step. 

### 3.7. Sorghum Bran Hydrolysis Using Crude Glucoamylase Solution

The utilisation of the crude glucoamylase for the hydrolysis of sorghum bran was carried out and compared with commercial enzymes (glucoamylase and α-amylase, from the Megazyme^@^ starch kit). The substrate loading ratio was 80 g/L, the enzyme loading ratio was 50 U/g dry weight sorghum bran and the hydrolysis was carried out at 55 °C for 120 h. By the end of the hydrolysis, the glucose concentration in the hydrolysis of the crude enzyme and the commercial enzyme were 11.32 ± 0.8 g/L and 11.74 ± 0.5 g/L, respectively, corresponding to a hydrolysis yield of 78.7% and 81.6% of the theoretical yield, respectively. In order to improve sugar content in the hydrolysate, a solid loading ratio of 200 g/L was carried out using a 2 L fermenter, at 55 °C, 500 rpm for 48 h. Around 700 mL of sorghum bran hydrolysate was obtained, with a glucose concentration of 38.7 ± 1.3 g/L, corresponding to 94.1% of the theoretical hydrolysis yield. 

### 3.8. Economic Evaluation of Glucoamylase Production in a Bioethanol Production Process

**Figure 7:**
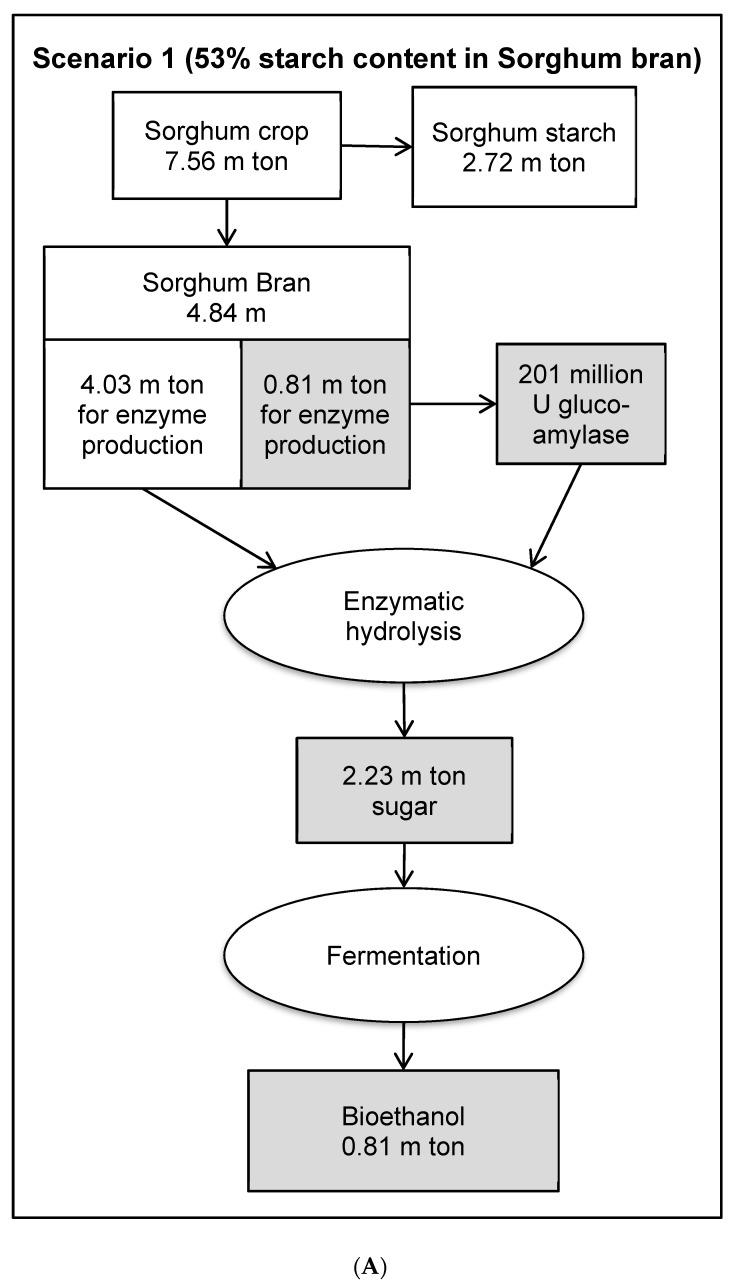

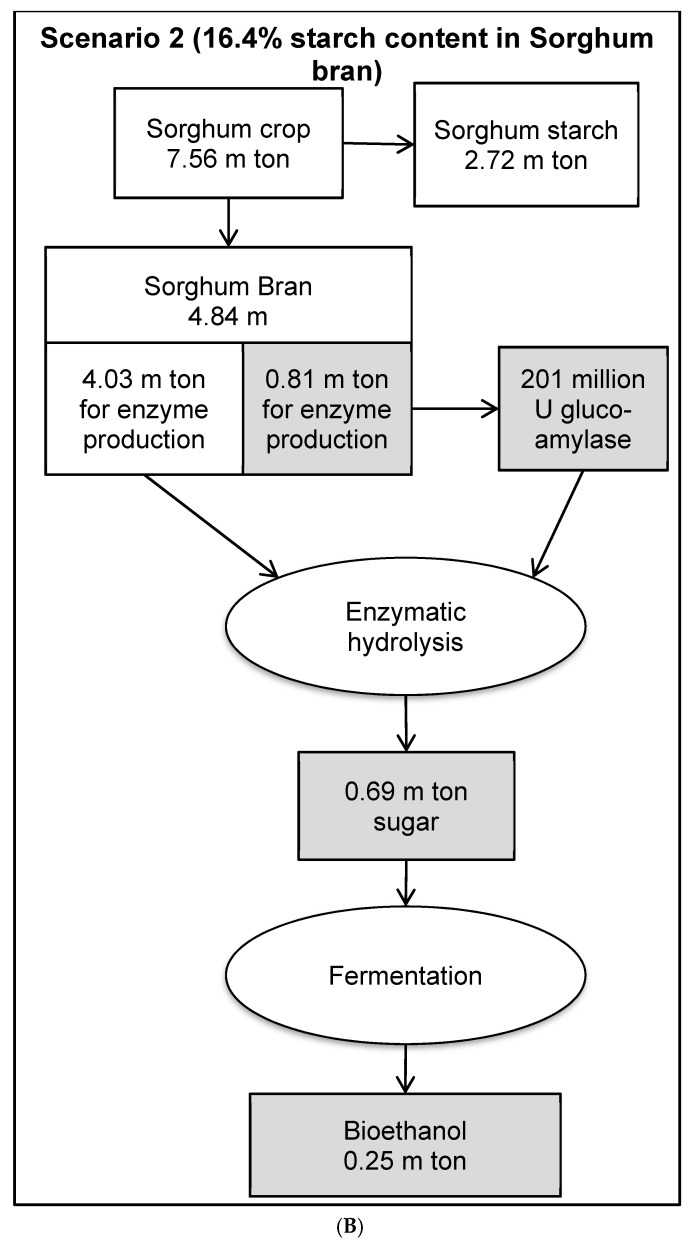
Mass balance of a sorghum bran based biorefining strategy for bioethanol production via glucoamylase hydrolysis process. (**A**): scenario 1, the starch content in sorghum bran was 53% [7]; (**B**) scenario 2, the starch content in sorghum bran was 16.4% (this study).

Ahmed El-Iman et al. recently developed a model for estimating bioethanol production potential in Nigeria [7]. In the model, an acid hydrolysis process was used, and the starch content in the sorghum bran was determined to be 52.96% (w/w). Some 0.73 million tons of bioethanol was estimated to be produced from 7.56 million tons of available sorghum crop [7]. If the acid hydrolysis process could be replaced by the integrated biorefining strategy reported in this study, a 10% increase in bioethanol production could be achieved (Figure 7A). As shown in Figure 7A, although 17% of the sorghum bran was used for glucoamylase production, the significant increase in hydrolysis yield (94.1%) led to 0.81 million tons of bioethanol being produced. This suggested that using sorghum bran for on-site glucoamylase hydrolysis would economically benefit the bioethanol production process. Inthe case that the sorghum bran contains 16.4% starch (Table 3), only 0.25 million tons of bioethanol could be produced (Figure 7B). In this scenario, it would be more economically feasible to use all available sorghum bran for glucoamylase production as enzymes are relatively higher value products than bioethanol, and the capital investigation for glucoamylase production would be lower than that for bioethanol production. 

## 4. Conclusions

In this study, the utilization of sorghum bran for the glucoamylase production was investigated. The compositional analysis indicated that sorghum bran derived from wet milling using a peanut butter maker contained 16.4% (w/w) starch. Investigation of fermentation conditions led to a 10-fold increase in glucoamylase production from 1.90 U/mL to 19.3 U/mL. Fermentations using 2-L fermenters confirmed the shake flask experimental results. Further increase in the substrate concentration to 12.5% (w/v) in 2-L fermentations achieved a glucoamylase concentration of 37.6 U/mL. The utilization of the enzyme solution for the hydrolysis of sorghum bran indicated that the crude enzyme was comparable with commercial enzymes. A sorghum bran hydrolysate containing 38.7 g/L glucose was obtained, which can be used as a generic fermentation feedstock for the fermentative production of biofuels and biochemicals. 

## Figures and Tables

**Table 1:** Recent studies on glucoamylase production via fungal fermentation.

Substrate	Strain	Fermentation Type	Gluco-Amylase Production	Ref.
Babassu cake (kernel residue)	*A. awamori*	SSF/4 days	22.8 U/mL	[16]
Babassu cake, castor seed, sunflower & canola cakes	*A. awamori, A. wenti*, *P. verrucosum*	SSF/4 days	29.8 U/g	[17]
Kitchen waste/Wheat bran	*A. niger*	SSF/5 days	1838 U/g	[18]
Pastry waste & mixed food waste	*A. awamori*	SSF/10 days	76.1 ± 6.1 U/mL	[19]
Rice bran	*A. awamori, A. niger, A. terreus, A. tamarii*	SmF	264.5 U/g	[20]
Sorghum pomace	*A. niger* and *Saccharomyces cerevisae*	SmF/3 days	3.3 U/mg protein	[21]
Waste bread	*A. awamori*	SSF/4–5 days	114–130.8 U/g	[22,23]
Waste potato mash	*A. niger*	SSF	274.4 U/mL	[24]
Wheat bran	*A. awamori*	SSF/4 days	9157 U/g	[25]
Wheat bran	*A. niger*	SSF/4 days	1.345 ± 0.009 IU/mL/min	[26]
Wheat milling by-product	*A. awamori*	SSF/4 days	48 U/g 4.4 U/mL	[27]

SSF: Solid state fermentation; SmF: Submerged fermentation; U/g: Unit per gram dry weight biomass.

**Table 2:** Mineral composition investigated in SmF.

Mineral Solution	Composition	REF
A	(NH_4_)_2_SO_4_ 1 g/L, KH_2_PO_4_ 0.5 g/L, K_2_HPO_4_ 0.5 g/L, MgSO_4_ 0.2 g/L	[32]
B	KH_2_PO_4_ 6 g/L, MgSO_4_·7H_2_O 1 g/L, FeCl_3_·4H_2_O 10 mg/L	[33]
C	KH_2_PO_4_ 1 g/L, MgSO_4_·7H_2_O 0.3 g/L, CaCl_2_ 0.3 g/L	[34]
D	KH_2_PO_4_ 1 g/L, MgSO_4_·7H_2_O 0.5 g/L	Designed in this study
E	NH_4_NO_3_ 1.5 g/L, K_2_HPO_4_ 2.5 g/L, KH_2_PO_4_ 1.5 g/L, MgSO_4_ 0.12 g/L, NaCl 0.25 g/L	Designed in this study

**Table 3:** The starch content in sorghum bran.

Sorghum species	Milling Processing	Starch Content (w/w)	REF
Red sorghum	Peanut butter maker	16.4 ± 1.3%	This study
Red sorghum	Blender	13.0 ± 0.8%	This study
Red sorghum	Knife mill	81.9 ± 3.2%	This study
Red sorghum	A tangential abrasive dehulling device	30%	[37]
Red sorghum	Buhler mill/hammer mill	24%	[4]
Red sorghum	Wet milling	52.96 ± 1.43%	[7]
White sorghum	Wet milling	49.7 ± 0.86%	[7]
